# Key Elements and Mechanisms of a Peer-Support Intervention to Reduce Loneliness and Isolation among Low-Income Older Adults: A Qualitative Implementation Science Study

**DOI:** 10.1177/07334648221120458

**Published:** 2022-09-02

**Authors:** Shannon M. Fuller, Ashwin A. Kotwal, Soe Han Tha, Daniel Hill, Carla Perissinotto, Janet J. Myers

**Affiliations:** 1 Division of Prevention Science, Department of Medicine, 8785 University of California San Francisco, San Francisco, CA, USA; 2 Division of Geriatrics, Department of Medicine, 8785 University of California, San Francisco, San Francisco, CA, USA; 3 Geriatrics, Palliative, and Extended Care Service Line, San Francisco Veterans Affairs Medical Center, San Francisco, CA, USA; 4 Curry Senior Center, San Francisco, CA, USA

**Keywords:** peer support, older adults, loneliness, qualitative methods, implementation science

## Abstract

This paper describes the evaluation of a longitudinal peer-support program developed to address loneliness and isolation among low-income, urban community-dwelling older adults in San Francisco. Our objective was to determine barriers, challenges, and successful strategies in implementation of the program. In-depth qualitative interviews with clients (*n* = 15) and peers (*n* = 6) were conducted and analyzed thematically by program component. We identified barriers and challenges to engagement and outlined strategies used to identify clients, match them with peers, and provide support to both peers and clients. We found that peers played a flexible, non-clinical role and were perceived as friends. Connections to community resources helped when clients needed additional support. We also documented creative strategies used to maintain inter-personal connections during the COVID-19 pandemic. This study fills a gap in understanding how a peer-support program can be designed to address loneliness and social isolation, particularly in low-income, urban settings.

**What this paper adds**

• Literature already shows that peer programs can be effective across a range of conditions—this paper unpacks the how and why a peer-support program can be highly impactful for older adults experiencing loneliness and isolation
• Details on the key mechanisms and components of a peer-support program to address loneliness and isolation among low-income older adults
• Perspectives from peers and clients involved in a peer-support program

**Applications of study findings**

• This study can inform the development, implementation, and scaling of other peer-support programs
• Key components of a successful peer-support program include thoughtful matching of peers and clients, flexible program design, ongoing supervision and training, and connection to community resources
• Our findings suggest that peer-support programs for low-income older adults are feasible to implement and highly acceptable to those involved

## Background

A growing body of evidence demonstrates an urgent need to develop programs addressing loneliness and isolation among older adults due to the broad impact these social factors can have on physical, psychological, and cognitive health (Bazari et al., 2018; Bruce et al., 2019). The National Academies of Sciences Engineering, & Medicine recently called for research to improve the understanding, prevention, and treatment of social isolation and loneliness in older adults across the country (NASEM, 2020).

Addressing loneliness among older adults can be complicated by other intersecting needs. Many at-risk older adults live alone, with disabilities, and below the federal poverty level (San Francisco Department of Aging and Adult Service, 2016), all of which are factors associated with higher risk of loneliness (Polenick et al, 2021). In 2015, the Curry Senior Center of San Francisco (hereafter, Curry) was awarded a 2-year contract by the Mental Health Services Act Oversight and Accountability Commission to develop and implement a peer-support program to address loneliness and isolation in the community.

Located in San Francisco’s Tenderloin neighborhood, Curry has been operating for over 45 years, providing holistic care and services for low-income, diverse older adults. The peer-support program was developed iteratively with guidance and experience from the community and staff at the center. The guiding theory was that a peer-support program may address complex, intersecting health and social needs by connecting individuals with shared life experiences. Although the program was developed without a specific theoretical underpinning, there is strong alignment in the academic literature around the value of peer support for behavioral health and loneliness (Lai et al., 2020; Theurer et al., 2021; Zeng & McNamara, 2021) as well as the importance of improving social skills and addressing maladaptive social cognition to address loneliness and isolation (Masi et al., 2011). A randomized study by Lai and colleagues in Canada found that a peer-support intervention reduced loneliness and isolation among older Chinese immigrants. In their conclusion, they called for further research to understand the effectiveness and delivery of peer-support programs. Our study aims to address this gap by using a qualitative implementation science approach to document the key components of a peer-support program and how those elements were experienced by peers and clients.

## Methods

To evaluate the peer-support program, in 2019, Curry partnered with our research team based at an academic medical center. Client outcomes are reported elsewhere (Kotwal et al., 2021). Using data from in-depth interviews, this study explores the barriers and challenges to implementation of the program, the strategies used to overcome them, and client and peer perspectives on program impact.

### Program Description

Program participants (“clients,” *n* = 74) were low-income, community-dwelling older adults (age 55 and older) of diverse racial and ethnic backgrounds recruited over a 2-year period. Many reported histories of homelessness and substance use. Clients were enrolled through Curry and other organizations known to serve older adults in the community. Peers (*n* = 8) were also diverse in terms of gender, sexual orientation, and racial/ethnic backgrounds. Similar to the clients, peers were older adults (55 and older) who had shared experiences such as living with loneliness, having experienced homelessness, and having histories of accessing behavioral health services.

**Figure 1.**
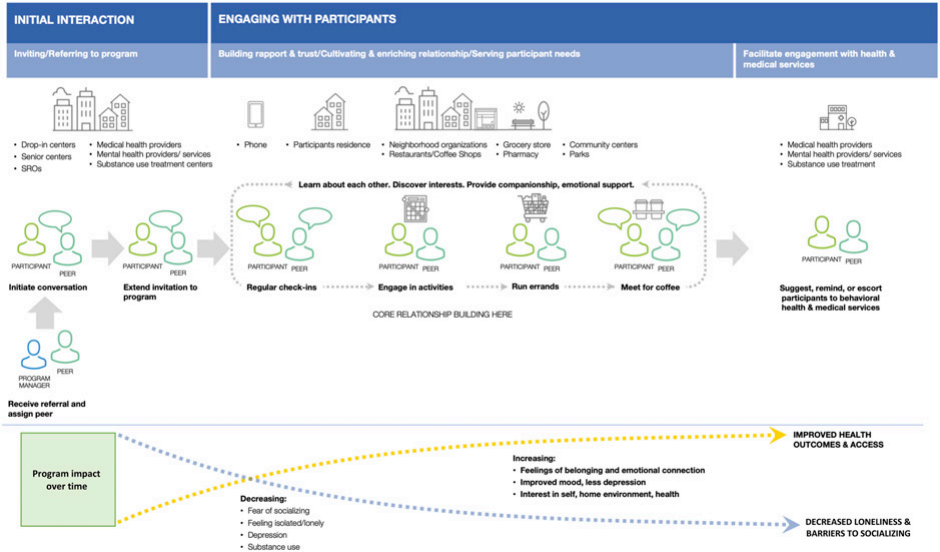
Understanding how clients are engaged and served by the peer-support program (“Journey Map”).

Figure 1 illustrates the components and how clients interact with the program (“journey map”). Peers completed a two-week initial training, a mental health certificate program, and additional monthly training sessions throughout the program. Once clients were matched to peers by the program manager, the peers and their clients typically met weekly, though the frequency and duration of visits varied depending on client preferences and needs. Peers also met weekly with each other and their supervisor to discuss challenges, strategies, and areas of need. Caseloads varied based on peer preference and capacity, ranging from 5 to 15 clients. The program was designed to ensure that peers had flexible, part-time work hours.

Recruitment into the program included the following steps: First, the program manager would receive a referral to people 55 and older from various community partners and clinicians from neighboring primary care clinics and related service settings. The program manager or one of the peers might also approach clients visiting the congregate meal program at Curry and would initiate a conversation with a potential program participant. If the person was interested, an invitation to the program would be extended and a participant would be matched to an appropriate peer. Matching was based on peer availability, common interests and on demographic characteristics, including gender, sexual orientation, and race/ethnicity, where possible. The peer would then conduct regular check-ins with the client and engage in activities with them related to common interests and emotional support needs. For example, peers would accompany clients on errands and meet them for social connections such as getting a cup of coffee. All activities were designed to increase feelings of belonging, emotional connection, and to improve mood and interest in social and health-related connections (Figure 1 and, for more detail, see Kotwal et al., 2021).

### Data Collection

For this analysis, semi-structured qualitative interviews were conducted with a sample of clients and peers. Clients were purposively sampled to represent diversity by gender and race/ethnicity. The program director worked with the peers to identify and facilitate recruitment of demographically diverse clients for phone-based interviews conducted by the evaluators (SF, JM). Interviews were conducted with 6 peers and 15 clients between March and June 2020 until thematic saturation was reached. Client interviewees included 10 men and 5 women, including 2 transgender women. Race/ethnicity reported by clients included 5 non-Hispanic White, 5 Latino/a, 2 African American, and 3 unknown. All clients were housed in single-room occupancy (SRO) housing in the San Francisco Tenderloin neighborhood. Demographic information for the 6 peers was reported as follows: 3 men and 3 women, including 1 transgender woman; and 2 non-Hispanic White, 2 Asian, 1 Latino/a, 1 Black/African American. All peers who were contacted consented to participate, and only one client declined due to scheduling conflicts. Participants had to be conversant in English or Spanish and willing and able to give informed consent. Clients received a gift card incentive ($20).

Interview guides were developed by the evaluators (SF, JM) for the peer and client interviews. Program leaders (CP, DH) reviewed the interview guides to ensure comprehensiveness and appropriateness. Interviews explored program experiences and perceived core elements, areas of unmet need, perceived impact of the program, and recommendations for improvements. Each interview lasted approximately 45 min. Interviews were audio-recorded and transcribed. Interviewers wrote field notes following each interview to summarize key points and capture emerging themes. This study was determined to be quality improvement by our university’s institutional review board.

In addition to conducting qualitative interviews, evaluators observed informal meetings of peers and supervisors and reviewed secondary documents such as progress reports and service manuals that explained the history of the program and how it evolved. Evaluators (SF, JM) wrote memos to complement the data collected through interviews and focused on documenting program design and other contextual information (Charmaz, 2007). We conducted our study in accord with the standards set out in the COREQ checklist (Tong et al., 2007; see supplementary material).

### Analysis

For analysis, we first conducted thematic analysis using a template technique to identify and refine themes in the interviews (Hamilton, 2013). Each transcript was reviewed as we prepared structured summary documents in Microsoft Word that organized the data into major topical areas: Challenges/Unmet Needs, Program Strategies and Program Impact. We also prepared and reviewed memos from our secondary document review and discussions with program leaders. All data were further consolidated into an analytic table that facilitated cross-case and within-case comparisons.

We then used an implementation science (IS) approach to understand how the topical areas of our analysis related to each other and could be used to inform the continuation and expansion of the program. Briefly, IS is the scientific study of methods to promote the systematic uptake of research findings and other evidence-based practices into routine practice to improve the quality and effectiveness of health services (for further reading, please see: Bauer et al., 2015). IS principally concerns processes related to program delivery (“implementation strategies;” Powell et al., 2015) and the contexts in which they are applied (“determinants” and, in this case, the barriers and facilitators of program success; Smith et al., 2020). Thus, the IS framework in study is not about determining the actual effectiveness of the program, as this has been published previously, but rather to guide our understanding of the implementation strategies, barriers, and facilitators, and implementation outcomes as reported by peers and clients.

## Results

In the sections below, we present the challenges that clients and peers experienced, and the main strategies used to overcome these challenges (Table 1). We also present clients’ and peers’ perceptions of program impact. Findings are organized below by each major component of program delivery.

**Table 1.** How the Intervention Addressed Challenges Related to Isolation, Loneliness, and Program Implementation.

Intervention component	Challenges and barriers	Strategies used to overcome challenges and barriers
Identifying, recruiting, and matching clients with peers	• Finding isolated clients	• Outreach to community organizations
	• Stigma from self-identifying as lonely or isolated	• Referrals from other organizations, service providers, and friends of clients.
	• Distrust or unfamiliarity with peers	• Direct outreach in group settings (e.g., lobbies of housing) “Soft approach” to initial visits
	• Difficulty recruiting diverse peers	• Broad recruitment strategy/hiring staff to reflect population being served
	• Small pool of peers	• Flexible matching process based on background, shared interests, and client preferences
		• Soft approach to recruiting clients and building rapport
Building rapport	• History of mistrust or elder abuse	• Identifying shared or unique interests (e.g., art, music)
	• Difficulty reaching homebound older adults or individuals with severe mental illness (e.g., depression)	• Discussing shared challenges (e.g., loneliness, mental health, homelessness, addiction)
		• Flexibility of visit schedule
Addressing barriers that contribute to isolation and loneliness connecting with services	• Maladaptive social cognition (e.g., loss of social skills or social anxiety)	• Providing opportunities for small group interactions (e.g., meals) to facilitate friendships and build confidence
	• Limited mobility; neighborhood safety	• Accompanying clients on walks or errands
	• Peers are not service providers	• Motivational interviewing, utilizing community resources
		• Suggesting, reminding, and accompanying clients to needed services
		• Reactivating existing relationships between clients and services
		• Coordination with caregivers
		• Sharing knowledge of available community resources
Maintenance of connection through program flexibility	• Maintaining boundaries with clients	• Ongoing training, supervision and mentorship
	• Unanticipated events (e.g., death of client, witnessed elder abuse, etc.)	• Responsive training sessions (e.g., grief counseling, harm reduction, aging education, diet and nutrition courses)
		• Regular collection and incorporation of feedback from peers and clients
		• Flexible design of program
Maintaining connections during COVID-19	• Limitations on in-person interactions	• Regular phone calls with clients. Continuing to share experiences, albeit virtually (e.g., watching the same show and then discussing over the phone, playing music together over the phone)

### Program Components

#### Identifying and Recruiting Clients and Matching them to Peers

For a peer-support program to be effective, identifying and recruiting clients is the first critical step. Program staff learned that recruiting from places where seniors already gathered (e.g., dining halls, senior centers) was helpful, but this meant that many people who were socially isolated were “invisible,” and those with safety or mobility concerns were similarly missed. Recruitment by flyer similarly had limited success because of the stigma associated with loneliness. Peers noted that few clients used the terms loneliness or isolation, though they might ask for support in ways that suggested they wanted companionship.

*Very few of them do [use the terms lonely or isolated]. Because I think it’s part of the stigma that nobody wants to say that they’re lonely… I can’t remember actually anybody saying that. Maybe it’s said in a different manner. For example, “You can always visit me.” I mean it’s sort of the same thing.*—Peer 02 (Note: Due to the small number of peers involved in the program, demographics are only reported in aggregate.)

To overcome recruitment challenges, the program manager gave presentations about the program at partner organizations. This facilitated client referrals from other organizations such as public health clinics and community centers that may have identified at-risk clients. Peers and program staff also directed outreach to building managers of local housing units in order to reach the “invisible.”

The second recruitment step involved matching peers and clients. This was central to the program and occurred at two levels: matching based on background and shared interests. To accomplish this, the program needed to recruit a diverse group of peers with the capacity to match clients based on language (English, Spanish, Cantonese), shared interests, or sexual orientation and gender identity. However, issues around perceived safety in the neighborhood presented difficulties recruiting peers, particularly women, so it was not always possible to match on all criteria. Matching based on shared interests was further facilitated by using open-ended questions when recruiting clients to gauge interests.

While demographic, cultural, or lifestyle concordance facilitated trust and bonding, it could also present challenges for maintaining boundaries. Additionally, occasionally shared experiences could bring up past trauma. Ongoing training and group meetings for the peers helped to address these concerns.

### Rapport Building

Peers used a “soft approach” when initiating contact with potential clients. A “soft approach” meant that while discussions might briefly introduce program goals to reduce loneliness and isolation, initial conversations typically followed a casual tone—it was simply an informal offer to visit and chat. A peer described this process in the quote below:

*I go and I introduce myself, and I tell them that I’m a peer outreach specialist. And I might even say my job is to help to reduce isolation and loneliness at the initial meeting... We don’t address it [loneliness and isolation] so much. It’s just understood. And then I tell them that we just have a friendly conversation. And I might help you with a few things. Or help you to help yourself, really.*—Peer 01

Most peers and clients found that rapport developed quickly, as the initial visit set a friendly, non-judgmental tone. Timelines for building closeness would vary from client to client. For example, a client who was blind and had previous experiences of people who took advantage of his disability required more time to develop a trusting relationship with the peer. In the quotes below, both the peer and client describe these initial hesitations:

*One of my clients I have is fairly new; he’s blind. I would visit him and take him to lunch and stuff… [At first] he didn’t trust me at all. And I understood why. He’s been blind, and people have ripped him off. So, he’s very suspicious of everyone…But then he started trusting me…I became the one that read his mail for him…. We built trust now.*—Peer 05

*When I first met him, I was a little hesitant. But I think that’s normal for first meetings. After that, it seems it’s been relaxed more. [Interviewer: All it took was that first meeting and you felt comfortable?] Yes. That’s always because for a blind person… or for anybody, you really have to feel each other out a little bit as far as what interests are and what you like doing.—*Client 09 (male, race/ethnicity unreported)

Nevertheless, a common theme was that clients could tell that the peers genuinely cared about them, which further promoted trust and bonding.

*[Interviewer: …when you get a new client, how long does it take to build trust with them?] For me, spontaneously…within the first month or so, within a few weeks. It just depends. I got ladies open up to me within the next week or so. Some it takes maybe a month … to warm up … [Interviewer: What makes you good at this work do you think?] Because I care. I care and they sense that, you know, and building care and trust, it builds rapport.”*—Peer 06

Shared experiences and common interests also fostered relationship-building and empathy.

*I look for their interests. If they’re a music lover, I look for programs that have music that they can enjoy or art. If they’re really into art, then I make plans to see an art exhibit or take them to a museum. Something that will interest them enough to get up in the morning or the afternoon and be looking forward to it. It’s just basically looking for their interests and going with that.*—Peer 05

*I’ve been through my own trials and tribulations just like they have, like everybody has. It’s easier to communicate that and to break the ice and to get through. It’s like I can say, “I know what you feel like. I’ve been there. And I’m not a doctor talking down to you, like, oh, you need this and that and here’s your problem because I say so.”*—Peer 03

### Addressing Barriers that Contribute to Isolation and Loneliness

As the program developed, it became clear that clients experienced common barriers to socializing, including neighborhood safety and co-existing mental health challenges. Over time, the program worked to address these barriers. One strategy involved adding group activities, including large groups (>10 persons) and smaller groups (<5) for clients who preferred a more intimate setting. These activities addressed the common barrier of maladaptive social cognition by helping participants gain confidence in socializing and allowing connection with people who shared similar interests. When asked about changes observed among clients, one of the peers talked about the benefit of the group programs:

*The clients that I’m working with are starting to build more friendships with each other. Five ladies have a circle now. Before the pandemic, there was a restaurant that we - Curry has a tab with. And that was part of our goal too, was to like connect people, connect them. Let’s take them there for lunch, get them socializing, if it’s okay with them of course… From what I’ve seen, having a peer come in and kind of push them a little bit in a gentle way and encourage them like, yeah, it’s okay to socialize still, you know, and you got it. You can stop, you got it. You know how to socialize, you know? The encouragement helps.*—Peer 06

When safety was identified as a barrier, clients appreciated having peers available to accompany them on walks and errands. Notably, many clients experienced violence or aggression in their neighborhood and had concerns about leaving their homes, particularly those with physical or visual impairment. Another client—a transgender woman who had experienced several instances of violence—felt comforted by the peer’s company.

*I know that I really trust [Peer]. I really know him very well. And I felt safer for him be go with to different places. Like so very happy – very secure.*—Client 07 (transgender woman, race/ethnicity unreported)

Crucially, peers recognized how symptoms of depression could cause clients to withdraw socially and that it may take extra time for them to engage in the program. Training in topics such as motivational interviewing and harm reduction helped peers learn how to work with some of the clients who were relatively harder to reach. In the quote below, a peer described the changes they had seen in one of their more isolated clients over time:

*There’s another one who was very isolated and didn't have much outside contact. We just started chatting on the phone and talking about different things. And we went over to [a community center] and we took in a movie. It used to be I would call and he wouldn’t answer the phone. But now he sees it's me and so he answers the phone. So I think seeing people kind of step out of depression a little bit, that’s the effect that I can see, that this is all happening.*—Peer 03

### Facilitating Connection with Other Services

When the program began, it was assumed that participants were not connected to service providers and that the peers should focus on connecting clients to services (e.g., health care, social service, case management, and behavioral health). In fact, this was one of the goals of the initial funding, to help “connect” isolated adults to services. However, peers discovered that many clients had connections, so their role mostly involved referring participants back to their providers by suggesting, reminding, or accompanying clients to appointments. Some clients experienced complex medical or functional needs that exceeded what peers could address. In these cases, peers would often collaborate with a home health aide or another caretaker if available to coordinate visits or errands. Peers also leveraged their knowledge of community resources to connect clients to additional services as needed. The program’s connection to Curry and its comprehensive array of services provided a valuable resource.

### Program Flexibility

The program’s flexibility and emphasis on avoiding a pre-determined agenda was one of its hallmarks. This approach facilitated trust and gave clients a safe space to build skills and confidence. It also allowed clients to be supported in ways that were most meaningful and impactful to them. For example, one client who had worked with two different peers (one who had since retired) described how they had gone so far as to help him set up an art show to display his work in a local café. When talking about how often he would see the peer, he said, “There’s no real strict formula that way for me with him – he’s just a friend.” Almost all participants used the word “friend” to describe the peer, and this dynamic felt unique compared to other programs.

*I don’t think of him as a care provider, more as like as for as a friend…. Well, he just came over. We found we had lots in common. And one thing led to another and we just talked a lot and enjoy each other’s sense of humor, I guess.*—Client 02 (White male)

As another client explained, having the peer as a friend rather than as a service provider was a welcome “break” and exactly what he needed:

*I like [the peer] as my friend. I don’t think about him as - here we got another counselor. I have already enough counselor. I need a break…I never feel like he’s working for me as my therapist or my counselor. I mean, I have therapists… Like I said, I look at [the peer] as my friend. I don’t consider him another caseworker. I don’t need no caseworker anymore. I have enough, I have enough of this. I need a friend.*—Client 04 (Middle Eastern/North African Male)

As noted above, the non-clinical role of the peer was critical as was flexibility. Yet, there were times where this flexibility could result in boundary crossing. Peers and clients also described challenges with emotional attachment, including coping with grief and loss. Connections to grief counseling and routine check-ins with the program supervisor helped, in addition to regular trainings and meetings with other peers.

*Last year, I had one client who passed away… I kind of saw that coming because he, you know, was not in good health. But after he passed away, I felt really bad, you know. I had to talk to [my supervisor]. And he asked me if you need to see a grief counselor, he can set up the appointment.*—Peer 04

### Maintaining Connection During the COVID-19 Pandemic

When shelter-in-place started, activities suddenly shifted from in-person to virtual. Peers were able to maintain connections with most clients during the pandemic, but a large part of that may have been influenced by pre-pandemic longstanding relationships and rapport. Peers described creative ways that they tried to maintain a connection with their clients, such as by watching the same television shows and then talking about them over the phone or by dropping off care packages. Even under the stressors introduced by the pandemic, the clients we interviewed felt that contacts by telephone were meaningful. Below, a client described how she valued the frequent check-ins by phone:

*Well, what has happened now, since this virus…He’d give me a call every day, check on me and see how I’m doing and just make sure I’m doing okay. He’s very caring to people. And, you know, I’m glad he calls. Because, you know, he’s part of my family, too.*—Client 07 (transgender woman, race/ethnicity unreported)

However, many clients also acknowledged that they were struggling with the additional isolation. For example, one client described how the experience has been difficult for her depression and recovery (“I’m not used to being in as much”)—but she appreciated how the peer recognized this challenge and validated her feelings.

## Discussion

Peer support has existed in a variety of behavioral health contexts (Mental Health America, n. d.; Zeng & McNamara, 2021). However, there is little if any literature on the implementation of peer-support programs to address loneliness. Through qualitative interviews, we uncovered challenges and key strategies associated with implementing a peer-support program to address loneliness and isolation among diverse, low-income older adults. Our implementation science approach highlights contextual factors that promoted successful engagement and outcomes from the program. Being flexible and adaptable, approaching individuals from a client-centered perspective and being aware of local community resources were key approaches. Informal, personal relationships were a hallmark of the program, as was the fact that this was peer-led and not counselor/caseworker-led. Our findings also showed the need for a structure to support peers and clients. Specifically, that structure involved thoughtfully matching peers and clients, providing ongoing training and supervision, facilitating group activities, and partnering with other community organizations.

Additional key lessons include the importance of understanding the population served—this involved understanding clients’ needs and how best to engage them—and having programmatic flexibility to meet needs as they arise. For example, the program reduced the age minimum from 60 to 55 years to account for the possibility of premature aging among low-income, homeless populations in San Francisco (Bazari et al., 2018; Patanwala et al., 2018). Although the clients lived in single-room occupancy housing, many had prior experiences of homelessness. Lowering the program’s age minimum allowed loneliness and isolation to be addressed in a population where the onset of aging is earlier.

Additionally, loneliness and social isolation are distinct yet related concepts and often require different strategies and interventions (Masi et al., 2011; NASEM, 2020). Several common themes in addressing loneliness emerged including building self-confidence, friendships, and a sense of belonging in the community. Strategies to reduce isolation involved creating opportunities for social connection by encouraging clients to attend gatherings and engage in community activities. The importance of flexibility in program development and implementation matches what is known about loneliness and isolation interventions—there cannot be a one size fits all approach. Accordingly, the program adapted over time to respond to several emerging needs. For example, peers incorporated more group-based activities and accompanied clients on errands to address safety concerns that were the underlying reasons for loneliness. The program also adapted its approach to support clients during the COVID-19 pandemic.

Our work adds to the growing body of literature on the mechanisms and potential benefits of peer-support programs (Theurer et al, 2021; Watson, 2019). A recent randomized-controlled study showed how phone calls that emphasized active listening reduced measures of loneliness, depression, and anxiety during COVID-19 (Kahlon et al., 2021). Although the individuals conducting these phone calls were not peers with the population being served, the study showed the benefit of a support program that was relatively non-structured and not explicitly goal-oriented. These findings also support what we documented during our interviews—phone calls can be an effective and acceptable delivery method, especially when there are restrictions with in-person contact. However, our interviews were conducted only a few months into the pandemic. Further study is needed to assess longer-term implementation of phone or video calls in providing social support for this population.

Most peer programs that address mental health tend to be time-limited, have formalized connections with medical professionals, and encourage clients to set specific goals during the course of the intervention (Chapin et al., 2013; Joo et al., 2016). This program was unique in the way that it was not time-limited—in part thanks to continued dedicated funding—and in the way that the program was driven by peers. Peers were able to be flexible in responding to what clients needed, and they felt appropriately supported and trained to do so. When clients had needs exceeding what peers could provide, the connections to community resources, particularly those at Curry, were essential.

Overall, our findings show that peer-support programs to reduce loneliness and isolation among low-income older adults can be feasible and acceptable. Our findings align with other implementation studies that have demonstrated the importance of organizational culture, training, and role clarification when integrating peer-support programs for mental health (Ibrahim et al., 2020; Mancini, 2018). We also found that supervision in addition to training helped support the peers and their work with clients, particularly when coping with emotionally challenging aspects of the job or trying to navigate boundaries.

As this program existed prior to and during COVID-19, it is important to note the effect of shelter-in-place on participants. Similar to other programs and organizations, in-person contacts were severely restricted for months. This led to changes in service delivery and posed new challenges with identifying and recruiting clients who may benefit from peer support.

Despite these limitations, the program was successful in its continued outreach efforts. Since COVID restrictions went into effect, referrals continued, however, several clients were lost to follow-up, primarily due to lack of access to technology and phones. This highlights the importance of recognizing the financial and digital divide that can further isolate those that are already vulnerable, which has also been noted in other recent studies (Polenick et al., 2021).

Our study has limitations. Due to the onset of the coronavirus pandemic, all data collection activities were moved from in-person to virtual. This limited our sample to those who had phones and sufficient minutes to participate in an interview. We attempted to mitigate this by coordinating with caregivers who could let the client borrow a phone for the interview. We also did not interview those who declined the program or dropped out, although we do have secondhand stories from the peers that can explain some of the reasons why some clients may have declined. Finally, this program focused on an urban, diverse and low-income population, and as such, its findings may need to be adapted for rural settings or areas with different demographics. However, our findings do correspond with other studies of peer-delivered loneliness interventions and add to a growing body of literature on their value (Lai et al., 2020; Theurer et al., 2021).

## Conclusions

This study demonstrates the feasibility and acceptability of a peer-support program to address loneliness and isolation and outlines several implementation challenges and strategies. Our findings can inform the design of future interventions to address loneliness and isolation, particularly among low-income older adults who experience complex and intersecting health and social needs.
